# Utilization of cardiopulmonary bypass at radical nephrectomy for renal cell carcinoma with tumour thrombus

**DOI:** 10.1002/bco2.460

**Published:** 2024-11-14

**Authors:** Chalairat Suk‐Ouichai, Mitchell M. Huang, Clayton Neill, Christopher K. Mehta, Ashley E. Ross, Shilajit D. Kundu, Kent T. Perry, Duc T. Pham, Hiten D. Patel

**Affiliations:** ^1^ Department of Urology, Feinberg School of Medicine Northwestern University Chicago Illinois USA; ^2^ Division of Cardiac Surgery, Department of Surgery, Feinberg School of Medicine Northwestern University Chicago Illinois USA

**Keywords:** cardiopulmonary bypass, IVC thrombus, nephrectomy, renal cell carcinoma, tumour thrombus

## Abstract

**Objectives:**

The objective of this study is to evaluate preoperative factors associated with cardiopulmonary bypass (CPB) utilization and outcomes for patients with renal cell carcinoma (RCC) and tumour thrombus (TT). Radical nephrectomy with thrombectomy is a standard treatment for patients with RCC and associated TT. Morbidity and mortality rates tend to correlate with aggressiveness of tumour and TT level.

**Methods:**

Patients undergoing radical nephrectomy with thrombectomy (2006–2023) were retrospectively identified. Inclusion criteria included RCC histology and preoperative imaging available for thrombus‐level categorization based on the Mayo Clinic grading system. Logistic regression assessed predictors for utilizing CPB, and Cox regression identified factors associated with survival.

**Results:**

A total of 72 patients with RCC and associated TT were identified. The median age was 67 years. RCC‐related symptoms were present in 83%, and 28% had Levels 3 and 4 thrombi. Eleven patients (15.3%) had undergone neoadjuvant therapy, and 81% had clear‐cell RCC. CPB was utilized in eight (11.1%) cases. The median tumour size was 10.5 cm. Metastatic disease was greater in the CPB cohort (75% vs. 28%, *p* = 0.008). All cases performed on CPB were Levels 3 and 4 thrombi (100% vs. 19% in the non‐CPB group, *p* < 0.001). CPB cases had significantly longer operative time, and hospital stays and rates of Clavien ≥ 3 complications. On multivariate analysis, metastatic disease was a predictor of CPB utilization. Median survival was 74 and 25 months in the non‐CPB and CPB cohorts, respectively (*p* = 0.01). Pulmonary disease and metastatic disease with CPB utilization were significantly associated with worse survival on multivariate analysis.

**Conclusions:**

Surgical extirpation of kidney tumours with associated TT remains the standard of care among patients with locally advanced RCC. CPB can be utilized to increase the feasibility of resection for high‐level thrombi. Preoperative planning and cooperation among surgical teams are key given the perioperative morbidity and mortality.

## 1 INTRODUCTION

Renal cell carcinoma (RCC) is a malignancy that exhibits the capacity to form a tumour thrombus (TT) and invade the venous system.^1^, ^2^, ^3^, ^4^ Approximately 10% of cases extend to renal vein and inferior vena cava (IVC), and 1% of cases progress to the right atrium.^1^, ^2^, ^3^, ^5^ Based on SEER data, patients who remained untreated had a significantly poor prognosis, with only 29% of them surviving beyond 1 year.^6^

Surgery is the most effective way to remove renal cancer and TT, but the procedures carry a high risk of morbidity and mortality.^1^, ^2^, ^4^, ^7^, ^8^ However, retrospective data from Mayo Clinic found that the early postoperative complication rate has decreased over time from 13.4% in 1970–1989 to 8.1% in 1990–2000, as well as perioperative mortality from 2.0% to 1.5%.^8^ Previous studies have shown a controversial association between high TT levels and worse survival, whereas local tumour invasion, involvement of lymph nodes and metastatic disease are predictors of poor prognosis after surgery.^5^, ^7^, ^9^, ^10^ Nephrectomy with tumour thrombectomy is a potential curative treatment for patients with non‐metastatic RCC.^2^, ^3^, ^4^ Surgical techniques, such as hepatic mobilization or venovenous bypass, have been introduced to avoid cardiopulmonary bypass (CPB) among patients with renal cancer and high TT levels to reduce the risk of complications associated with, such as platelet dysfunction, coagulopathy caused by intraoperative heparinization and neurological complications.^2^, ^3^, ^11^, ^12^, ^13^ Controversial survival benefits associated with the utilization of CPB have been discussed, and no predictors for CPB utilization have been identified among patients with renal cancer and TT.^3^, ^14^

We conducted a study to identify preoperative factors associated with the use of CPB and determine how these factors impact the survival outcomes of RCC and TT patients. Perioperative outcomes and complications related to the use of CPB were also evaluated.

## 2 METHODS

Patients with a renal mass and associated TT were identified from the Northwestern Medical Group Electronic Data Warehouse from 2006 to 2023. All patients who underwent radical nephrectomy with tumour thrombectomy were retrospectively reviewed (*n* = 79). Only patients with RCC histology were included (*n* = 72). All patients had preoperative CT or MRI images available to categorize thrombus level using the Mayo Clinic grading system.^15^

Patient demographics, including age, gender, race, body mass index (BMI), renal function, presenting symptoms and comorbidities, and tumour characteristic information, were collected. The surgical approaches, TT removal technique and decision on CPB utilization were based on the surgeon's decision. The consultation of cardiovascular and thoracic surgeons was initiated upon diagnosis of RCC with TT. The decision of CPB utilization was based on the extension of TT intraoperatively and the ability to completely remove TT. Perioperative data were recorded as operative time, estimated blood loss (EBL), length of stay (LOS) and complications. Postoperative complications within 30 days were classified based upon the Clavien–Dindo classification.^16^ Patients were surveyed postoperatively by cross‐sectional images at 3 months and then every 3–6 months regarding findings. Overall survival was defined from the surgery date to the last follow‐up or death.

### 2.1 Statistical analysis

The analyses were performed with Stata version 18 (TX: StataCorp LLC). Continuous variables were shown as median (interquartile range [IQR]), and categorical variables were in numbers (percentages). Chi‐square and Mann–Whitney *U* tests were performed to compare CPB and non‐CPB cohorts. Kaplan–Meier curve demonstrated the survival graphs, and log‐rank test was performed to compare the outcomes. Logistic regression was performed to assess the predictors for utilizing CPB and Cox regression for factors associated with overall survival (OS). Variables with a *p* value of ≤0.3 in univariate analysis were further analysed in multivariate regression. All statistical significance was considered at a *p* value of <0.05.

## 3 RESULTS

### 3.1 Patient and tumour characteristics

Of 72 RCC patients with TT, the median age was 67 years, and 65% were male (Table 1). The cohort was mainly composed of Caucasians (68.1%), followed by Hispanics (11.1%) and African Americans (8.3%). The median BMI was 28.3 kg/m^2^. Sixty patients (83%) were symptomatic at presentation. Among the patients, hypertension was the most prevalent comorbidity, affecting 82% of them (59 patients). Additionally, 30 patients (42%) had coronary artery disease (CAD), 22 patients (31%) had pulmonary disease, 26 patients (36%) had liver disease, 14 patients (19%) had peripheral vascular disease, 44 patients (61%) had dyslipidemia, and 34 patients (47%) had diabetes. The median preoperative and postoperative eGFR were 61 and 55 ml/min/1.73 m^2^, respectively.

**TABLE 1:** Patient and tumour characteristics

	All patients (*n* = 72)	Patients not using CPB (*n* = 64)	Patients utilizing CPB (*n* = 8)	*p* value
Age (years), median (IQR)	67 (60–76)	68 (59–77)	63 (62–65)	0.26
Male, *n* (%)	47 (65.3)	43 (67.2)	4 (50.0)	0.34
Race, *n* (%)				0.40
Caucasian	49 (68.1)	43 (67.2)	6 (75.0)	
African American	6 (8.3)	6 (9.4)	0 (0)	
Hispanic	8 (11.1)	8 (12.5)	0 (0)	
Others	9 (12.5)	7 (10.9)	2 (25.0)	
BMI (kg/m^2^), median (IQR)	28.3 (24.7–32.5)	27.9 (24.6–32.5)	30.8 (27.9–34.1)	0.25
Symptoms at presentation, *n* (%)	60 (83.3)	53 (82.8)	7 (87.5)	0.74
Coronary artery disease, *n* (%)	30 (41.7)	25 (39.1)	5 (62.5)	0.21
Pulmonary disease, *n* (%)	22 (30.6)	21 (32.8)	1 (12.5)	0.24
Liver disease, *n* (%)	26 (36.1)	24 (37.5)	2 (25.0)	0.49
Peripheral vascular disease, n (%)	14 (19.4)	14 (21.9)	0 (0)	0.14
Hypertension, *n* (%)	59 (81.9)	53 (82.8)	6 (75.0)	0.59
Dyslipidemia, *n* (%)	44 (61.1)	40 (62.5)	4 (50.0)	0.49
Diabetes, *n* (%)	34 (47.2)	31 (48.4)	3 (37.5)	0.56
Chronic kidney disease, *n* (%)	39 (54.2)	35 (54.7)	4 (50.0)	0.80
Preoperative eGFR, median (IQR)	61 (50–77)	62 (49–79)	58 (52–76)	0.68
Postoperative eGFR, median (IQR)	55 (45–71)	55 (45–70)	52 (40–92)	0.90
Tumour size (cm), median (IQR)	10.5 (8.0–13.5)	10.5 (8.3–13.5)	8.95 (5.8–12.5)	0.17
Right kidney, *n* (%)	54 (75.0)	49 (76.6)	5 (62.5)	0.39
Thrombus level, *n* (%)				***<0.001***
Level 0	12 (16.7)	12 (18.8)	0 (0)	
Level 1	12 (16.7)	12 (18.8)	0 (0)	
Level 2	28 (38.9)	28 (43.8)	0 (0)	
Level 3	12 (16.7)	9 (14.1)	3 (37.5)	
Level 4	8 (11.1)	3 (4.7)	5 (62.5)	
Neoadjuvant treatment, *n* (%)	11 (15.3)	9 (14.1)	2 (25.0)	0.42
Metastatic disease, *n* (%)	24 (33.3)	18 (28.1)	6 (75.0)	***0.008***
Renal cell carcinoma, *n* (%)				0.74
Clear cell	58 (80.6)	51 (79.7)	7 (87.5)	
Papillary	6 (8.3)	5 (7.8)	1 (12.5)	
Xp11 translocation	3 (4.2)	3 (4.7)	0 (0)	
Unclassified	5 (6.9)	5 (7.8)	0 (0)	
Tumour necrosis, *n* (%)	46 (63.9)	40 (62.5)	6 (75.0)	0.49
Sarcomatoid, *n* (%)	6 (8.3)	5 (7.8)	1 (12.5)	0.65
Rhabdoid, *n* (%)	9 (12.5)	8 (12.5)	1 (12.5)	0.10
Operative time (minutes), median (IQR)	279 (167–398)	264 (156–345)	518 (445–583)	***<0.001***
Estimated blood loss (ml), median (IQR)	1125 (525–2400)	1025 (525–2150)	2345 (1250–3250)	0.10
Length of hospital stay (days), median (IQR)	6 (4–9)	5 (4–8)	18 (10–25)	***0.006***
Grade 3 Clavien classification or above, *n* (%)	10 (13.9)	6 (9.4)	4 (50)	***0.002***
Median follow‐up (months)	19.7	20.0	6.3	0.05
Median survival (months)	74.4	74.4	24.5	***0.01***
2‐year overall survival	83.0%	85.2%	62.5%	
5‐year overall survival	68.3%	72.0%	0%	

*Note*: Bold and italics‐bold text show statistical significance.

The median tumour size was 10.5 cm. There were 12, 12, 28, 12, and 8 patients with TT levels 0–4, respectively (Table 1). Eleven patients (15%) received neoadjuvant systemic treatment, and 24 patients (33%) had metastasis. The primary histology was clear cell RCC (81%), followed by papillary (8%), unclassified (7%), and Xp11 translocation (4%) RCC. The median operative times were 279 min. The median EBL was 1.13 L, and the median LOS was 6 days. There was no perioperative mortality, and the major complication (≥grade 3 Clavien) rate was 14%.

### 3.2 Comparison between CPB and non‐CPB cohorts

In the study, 64 patients were in the non‐CPB cohort and 8 patients in the CPB cohort (Table 1). The CPB cohort's patients were slightly younger than the non‐CPB group (63 vs. 68 years). The median BMI and percentage of symptomatic RCC were comparable between cohorts. The prevalence of comorbidities was also similar between the two cohorts. The preoperative and postoperative eGFR of both cohorts were not significantly different.

The primary tumour size of patients utilizing CPB was slightly smaller than those not using CPB but not statistically significant (8.95 cm vs. 10.5 cm, *p* = 0.17). The patients in the CPB cohort had significantly higher TT levels compared to those in the non‐CPB cohort (*p* < 0.001). Of the eight patients in the CPB cohort, five patients had Level 4 TT, and the others had Level 3 TT. Three patients with Level 4 TT had their thrombus manipulated down below the diaphragm intraoperatively and removed without utilizing CPB. Three out of 12 patients with Level 3 TT underwent CPB in unplanned fashion. The reasons were due to pulmonary embolism, hemodynamic instability requiring return visit to operating room for resection, and dislodged TT. Deep hypothermic circulatory arrest (DHCA) was used in four (50%) out of eight cases in the CPB cohort.

CPB‐utilizing patients tended to receive neoadjuvant treatment; however, it was not statistically significant (25% vs. 14%, *p* = 0.42). More patients in CPB cohort had metastatic RCC compared to non‐CPB cohort (75% vs. 28%, *p* = 0.008). Clear cell RCC was a common pathology in both cohorts. CPB patients had significantly longer operative time (518 vs. 264 min, *p* < 0.001) and LOS (18 vs. 5 days, *p* = 0.003). Although CPB patients had greater EBL, it was not statistically significant (2.35 vs. 1.03 L, *p* = 0.10). When comparing perioperative parameters among Levels 3 and 4 TT (Table S1), only operative time was significantly greater in the CPB cohort (518 vs. 291 min, *p* = 0.004). EBL and LOS were greater in CPB cohort but not statistically significant.

The rate of major complications was notably higher in the CPB group compared to the non‐CPB group (50% vs. 9%, *p* = 0.002). Of the four patients utilizing CPB, two of them had pulmonary emboli, one required thoracocentesis, and one had delayed abdominal closure. Among the six patients in the non‐CPB group, one had pulmonary embolism, two had perihepatic hematomas, one had hemoperitoneum, one had chylous ascites which required paracentesis, and one had a clip on the contralateral UPJ. When limited to the subgroup of patients with high TT (Levels 3 and 4), there was no significant difference in major complications between two cohorts (Table S1).

### 3.3 Factors associated with CPB utilization

In Table 2, univariate analysis showed that metastatic disease was significantly associated with the utilization of CPB (OR 7.67, 95% CI 1.41–41.6). This association remained significant after multivariate analysis (OR 41.4, 95% CI 3.34–513.2). Other factors were not statistically significant. When the analysis focused on the limited sample of patients with high TT levels (*N* = 20), there was no factor significantly associated with the use of CPB (Table S2).

**TABLE 2:** Univariate and multivariate analyses for utilizing cardiopulmonary bypass

	Univariate analysis		Multivariate analysis	
	OR (95% CI)	*p* value	OR (95% CI)	*p* value
Age	0.97 (0.90–1.03)	0.33		
BMI	1.10 (0.94–1.28)	0.22	1.18 (0.95–1.46)	0.13
Symptom at presentation	1.45 (0.16–13.0)	0.74		
Coronary artery disease	2.60 (0.57–11.9)	0.22	2.58 (0.39–17.0)	0.33
Pulmonary disease	0.29 (0.03–2.53)	0.27	0.08 (0.00–1.17)	0.07
Preoperative eGFR	0.99 (0.95–1.02)	0.47		
Tumour size (cm)	0.86 (0.69–1.07)	0.17	0.77 (0.58–1.01)	0.06
Neoadjuvant treatment	2.04 (0.35–11.7)	0.43		
Metastatic disease	7.67 (1.41–41.6)	***0.018***	41.4 (3.34–513.2)	***0.004***

*Note*: Bold and italics‐bold text show statistical significance.

### 3.4 Survival outcomes

**FIGURE 1:**
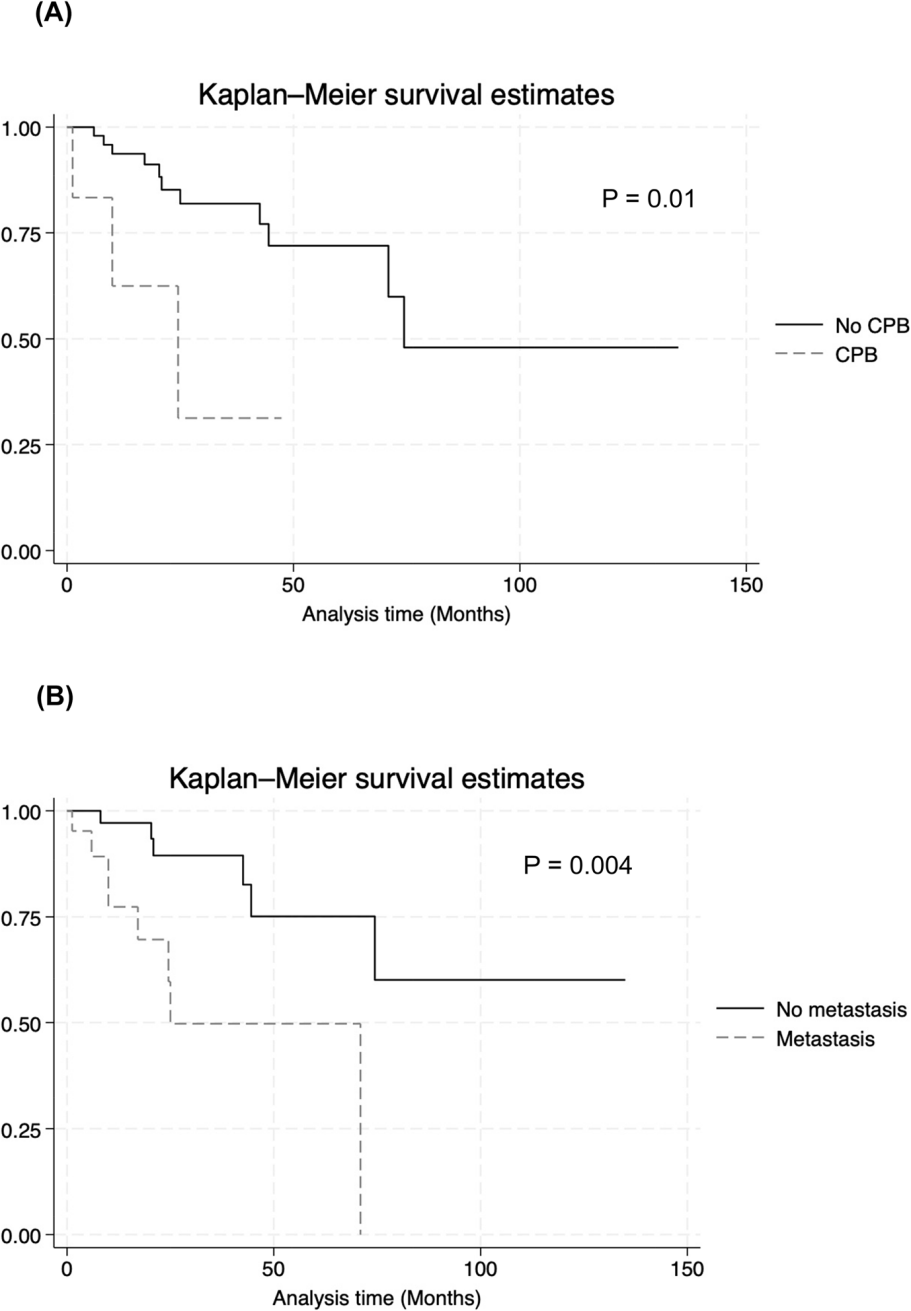
Kaplan–Meier survival estimates comparing overall survival of (A) patients who utilized cardiopulmonary bypass (CPB, represented by a dashed line) to those who did not use CPB (represented by a solid line) and (B) patients with metastases (dashed line) to patients without metastases (solid line). A log‐rank test was performed to compare overall survival outcomes.

The median follow‐up time was 19.7 months: 20 and 6.3 months in non‐CPB and CPB cohorts, respectively (*p* = 0.05). The median OS was 74.4 months for all RCC with TT patients. The median survival OS of CPB cohort was significantly lower compared to non‐CPB cohort (*p* = 0.01, Figure 1A). The non‐CPB cohort had a 2‐year OS of 85% and a 5‐year OS of 72%, while the CPB cohort had a 2‐year OS of 63% and a 5‐year OS of 0%. When comparing patients who had high TT, there was no significant difference in median survival between 2 cohorts (Table S1 and Figure S1).

Patients with metastases also had worse OS compared to patients without metastases (Figure 1B). The 2‐ and 5‐year OS of non‐metastatic patients was 90% and 75%, respectively. The 2‐ and 5‐year OS of patients with metastases was 70% and 50%, respectively.

On univariate analysis, BMI, pulmonary disease, metastatic disease, and utilizing CPB were found to be associated with OS (Table 3). Pulmonary disease and the use of CPB were significantly associated with worse OS on multivariate analysis. Given that metastatic disease was associated with CPB utilization, the interaction term of these factors was also assessed. Patients with metastasis and undergoing CPB utilization were significantly associated with worse OS on univariate (*p* = 0.004) and multivariate analyses (*p* < 0.001).

**TABLE 3:** Factors associated with overall survival among RCC with TT patients

	Univariate analysis		Multivariate analysis	
	HR (95% CI)	*p* value	HR (95% CI)	*p* value
Age	1.01 (0.97–1.06)	0.57		
BMI	0.86 (0.76–0.97)	***0.01***	0.88 (0.78–1.01)	0.06
Symptom at presentation	1.02 (0.31–3.37)	0.98		
Coronary artery disease	1.09 (0.38–3.16)	0.87		
Pulmonary disease	4.96 (1.59–15.4)	***0.006***	6.13 (1.39–26.9)	***0.016***
Preoperative eGFR	1.00 (0.98–1.03)	0.90		
Postoperative eGFR	1.01 (0.98–1.04)	0.36		
Tumour size (cm)	0.98 (0.83–1.14)	0.77		
Tumour thrombus level 3 or 4	1.67 (0.57–4.88)	0.35		
Neoadjuvant treatment	0.79 (0.10–6.14)	0.82		
Metastatic disease	4.51 (1.46–13.9)	***0.009***	2.33 (0.68–7.98)	0.18
Cardiopulmonary bypass (CPB)	4.64 (1.25–17.3)	***0.02***	12.1 (1.66–88.5)	***0.014***
Tumour necrosis	3.17 (0.87–11.6)	0.08	2.05 (0.44–9.48)	0.36
Sarcomatoid	0.83 (0.11–6.47)	0.86		
Rhabdoid	0.91 (0.11–7.05)	0.93		

*Note*: The study assessed the interaction term of metastatic disease and CPB utilization, finding a significant association with worse overall survival in both univariate (*p* = 0.004) and multivariate (*p* < 0.001) analyses. Bold and italics‐bold text show statistical significance.

## 4 DISCUSSION

Radical nephrectomy with tumour thrombectomy is the most effective treatment for patients with RCC and TT in both metastatic and non‐metastatic conditions.^3^, ^4^, ^5^, ^9^, ^17^ In the old immunotherapy era, metastatic RCC with TT patients who underwent surgery demonstrated comparable responses with those without TT.^5^ Our study found that the 5‐year OS rate of patients with metastatic RCC and TT after surgery was 50% while it was 75% for patients with non‐metastatic disease.

Collaboration between surgical teams, such as urologists, transplant specialists, and cardiovascular and thoracic surgeons, is critical to achieving success. In order to achieve a bloodless operative field, CPB has been utilized among patients with high TT levels for meticulous thrombus removal. Our study found that metastatic RCC was associated with CPB utilization. A previous study of 362 patients with RCC and Levels 3 and 4 TT from 22 US and European centres (1992–2012) who underwent surgery did not find a significant difference in survival outcomes between CPB and non‐CPB cohorts.^14^ The median OS was 24.6 and 29.3 months in non‐CPB and CPB cohorts, respectively. Lymph node involvement and metastatic disease were significantly associated with OS. Although the OS between CPB and non‐CPB cohorts showed a significant difference (*p* = 0.01) in our study, patients with Levels 3 and 4 TT had comparable median survival in the CPB and non‐CPB cohorts (*p* = 0.09).

Although morbidity and mortality have decreased over time, it is still a cause for concern of nephrectomy with tumour thrombectomy, especially in high‐level TT patients.^1^, ^8^, ^9^, ^18^, ^19^ The retrospective review of 427 metastatic RCC with TT patients who underwent surgery between 2000 to 2014 at 5 tertiary centres in the United States reported 10% 90‐day perioperative mortality and 4% occurred in the first 30 days.^17^ The median OS was 18.9 months, and the predictors of worse OS were elevated serum lactate dehydrogenase, systemic symptoms, supradiaphragmatic IVC thrombus, and sarcomatoid dedifferentiation. Our study demonstrated that pulmonary disease and metastatic disease patients who underwent CPB surgery were associated with worse OS among RCC with TT patients. This might be because pulmonary metastasis is common in RCC and patients with lung disease have compromised pulmonary reserve. Patients with metastatic disease and who had CBP surgery tend to have a high cancer burden. Therefore, OS was poor for patients with such conditions.

The use of CPB during surgery can result in postoperative complications, including hemorrhagic and neurological complications, which remain significant concerns.^3^, ^13^ The topic of whether using CPB leads to complications or not is a matter of controversy and debate.^14^, ^20^, ^21^, ^22^ A retrospective study of high‐level TT patients from four tertiary centres in the United States between 2000 and 2013 found no significant association between CPB utilization and postoperative major complications within 90 days (HR1.93, 95% CI 0.93–4.01).^21^ The overall and major complication rates were higher in the non‐CPB cohort compared to the CPB cohort from 22 US and European centres (overall: 60% vs. 32%, and major: 28% vs. 23%).^14^ Our study demonstrated significantly greater major complications in the CPB cohort compared to the non‐CPB cohort (50% vs. 9%); however, there was no significant difference when comparing among levels 3 and 4 TT patients.

Other surgical approaches have been introduced to avoid CPB utilization among patients with high‐level TT, which is associated with perioperative complications.^11^, ^12^, ^13^, ^19^ DHCA, in addition to CPB, was reported in one study to be associated with decreased perioperative mortality among patients with level 4 TT (HR0.13, 95% CI 0.04–0.5).^11^ Compared to the CPB‐only cohort, the DHCA cohort had significantly lower perioperative mortality rates (8.3% vs. 37.5%; *p* = 0.006). In our cohort, cardiothoracic surgeons generally considered DHCA when emboli to the lungs were suspected, and there was a need to stop circulation to adequately visualize and extract the thrombus/tumour. Ciancio et al.^12^ from the University of Miami proposed a piggyback‐style approach for liver mobilization to expose the upper retroperitoneal space entirely. The approach facilitates the mobilization of some non‐adherent thrombus within the intrapericardial IVC without sternotomy or thoracoabdominal approach. Out of 12 supradiaphragmatic TT patients, two died during the perioperative period: one due to arrhythmia and the other from acute respiratory distress syndrome. In our study, three out of eight patients with Level 4 TT underwent thrombectomy without CPB by milking down the thrombus below the diaphragm.

Of 72 patients involved in our study, 11 of them (15.3%) underwent neoadjuvant systemic treatment in order to reduce the cancer burden. Neoadjuvant therapy generally aims to facilitate complete resection in complex cases and prevent injury to surrounding organs. Previous studies on neoadjuvant targeted therapy among RCC with TT patients demonstrated limited clinical benefits. The response rate was reported at 22–44% after conventional targeted therapy; however, the change in TT level was small and unpredictable.^2^, ^23^, ^24^ A multicentre retrospective review of 19 patients with RCC and TT Levels 1–3 who received neoadjuvant sunitinib demonstrated that 42% of the patients experienced TT level reduction with a median reduction of 1.3 cm of TT size.^25^ Additionally, neoadjuvant sunitinib was significantly associated with improved cancer‐specific survival compared to those with primary surgery. The recent Phase II single‐arm, multicentre study of neoadjuvant axitinib in 20 patients with RCC and TT reported a 35% reduction in TT level and a 75% reduction in TT length. Neoadjuvant stereotactic radiation of TT has been administered; however, the benefits on operative and survival outcomes were not significantly found.^26^, ^27^ Clinical trials are ongoing for immune checkpoint inhibitors and new targeted therapy in patients with RCC and TT.

Our study has some limitations due to its retrospective nature and the fact that the data was retrieved from a tertiary centre. Additionally, the number of patients included in the study is limited due to the small prevalence of the disease. Some informative data that would have been useful for survival analysis is also lacking. However, the study had a moderate follow‐up time, and the number of patients was sufficient for reasonable analysis. The results of our study can help improve the care and treatment of patients with RCC and TT by providing valuable insights into the use of CPB and its impact on patient outcomes.

## 5 CONCLUSION

The standard of care for patients with locally advanced kidney cancer with associated TT is surgical removal. Radical nephrectomy with tumour thrombectomy is associated with notable morbidity. CPB can be used to make resection of high‐level thrombi more feasible. To reduce perioperative morbidity and mortality, preoperative planning and cooperation among surgical teams are essential practices.

## AUTHOR CONTRIBUTIONS

**Chalairat Suk‐Ouichai:** Conceptualization; data curation; formal analysis; project administration; writing—original draft preparation. **Clayton Neill:** Resources. **Mitchell M. Huang**, **Christopher K. Mehta**, **Ashley E. Ross**, **Shilajit D. Kundu**, **Kent T. Perry Jr.**, and **Duc T. Pham:** Methodology; resources; writing—review and editing; supervision. **Hiten D. Patel:** Conceptualization; methodology; resources; writing—review and editing; supervision.

## CONFLICT OF INTEREST STATEMENT

None of the authors have any disclosures or conflict of interest to report.

## Supporting information


**Figure S1.** Kaplan‐Meier survival estimates among patients with level 3‐4 tumor thrombi comparing the overall survival of patients who utilized cardiopulmonary bypass (CPB, represented by a dashed line) to those who did not use CPB (represented by a solid line). A log‐rank test was performed to compare overall survival outcome.

**Table S1** Patients with tumor thrombus level 3 or 4.

**Table S2** Univariate and multivariate analyses for utilizing cardiopulmonary bypass among patients with thrombus level 3‐4.
