# Supplementary figures and images for: Utilization of cardiopulmonary bypass at radical nephrectomy for renal cell carcinoma with tumour thrombus
Source: BJUI Compass. 2024 Nov 14;6(1):e460. doi: 10.1002/bco2.460 (PMC11771504; doi:10.1002/bco2.460)

**Supplementary Figure 1**


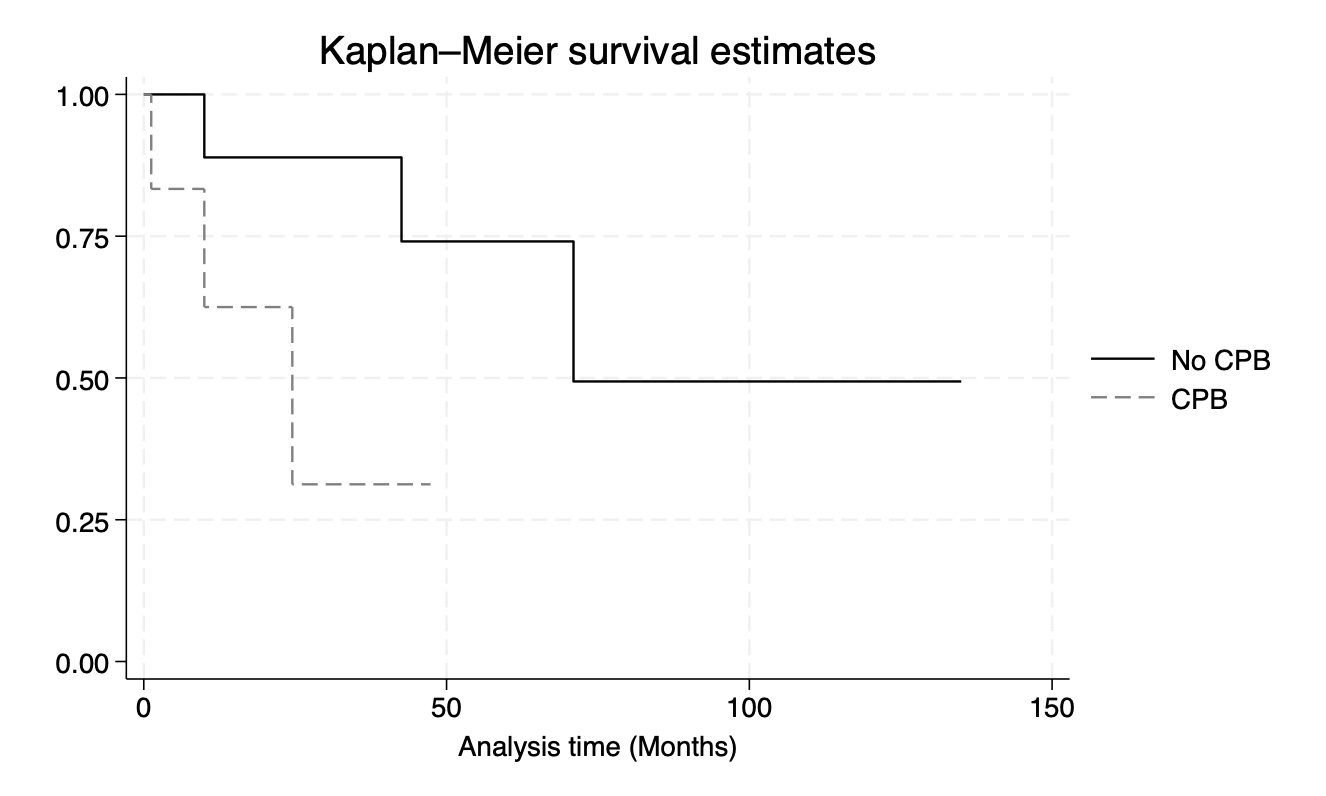


P = 0.09

Supplement: Supplementary file 1 — Figure S1. Kaplan‐Meier survival estimates among patients with level 3‐4 tumor thrombi comparing the overall survival of patients who utilized cardiopulmonary bypass (CPB, represented by a dashed line) to those who did not use CPB (represented by a solid line). A log‐rank test was performed to compare overall survival outcome. [file BCO2-6-e460-s001.docx]
