# Supplementary material for: Utilization of cardiopulmonary bypass at radical nephrectomy for renal cell carcinoma with tumour thrombus
Source: BJUI Compass. 2024 Nov 14;6(1):e460. doi: 10.1002/bco2.460 (PMC11771504; doi:10.1002/bco2.460)
Supplement: Supplementary file 2 — Table S1 Patients with tumor thrombus level 3 or 4. [file BCO2-6-e460-s003.docx]

**Supplementary Table 1** Patients with tumor thrombus level 3 or 4

|  | **All patients (n=20)** | **Patients not using CPB (n=12)** | **Patients utilizing CPB (n=8)** | **p-value** |
| --- | --- | --- | --- | --- |
| Operative time (minutes), median (IQR) | 400 (279-502) | 291 (264-404) | 518 (445-583) | ***0.004*** |
| Estimated blood loss (ml), median (IQR) | 2050 (500-3000) | 2000 (500-2900) | 2345 (1250-3250) | 0.54 |
| Length of hospital stay (days), median (IQR) | 10 (6-25) | 9 (5-20) | 18 (10-25) | 0.31 |
| Grade 3 Clavien classification or above, n (%) | 6 (30.0) | 2 (16.7) | 4 (50) | 0.11 |
| Median follow-up (months) | 15.8 | 38.6 | 6.3 | 0.07 |
| Median survival (months) | 71.0 | 70.1 | 24.5 | 0.09 |
