# Supplementary material for: Utilization of cardiopulmonary bypass at radical nephrectomy for renal cell carcinoma with tumour thrombus
Source: BJUI Compass. 2024 Nov 14;6(1):e460. doi: 10.1002/bco2.460 (PMC11771504; doi:10.1002/bco2.460)
Supplement: Supplementary file 3 — Table S2 Univariate and multivariate analyses for utilizing cardiopulmonary bypass among patients with thrombus level 3‐4. [file BCO2-6-e460-s002.docx]

**Supplementary Table 2** Univariate and multivariate analyses for utilizing cardiopulmonary bypass among patients with thrombus level 3-4

|  | **Univariate analysis** | | **Multivariate analysis** | | |
| --- | --- | --- | --- | --- | --- |
|  | OR (95% CI) | p-value | OR (95% CI) | p-value |  |
| Age | 0.73 (0.50-1.05) | 0.09 | 0.69 (0.45-1.07) | 0.10 |  |
| BMI | 1.07 (0.90-1.27) | 0.43 |  |  |  |
| Symptom at presentation | 1.40 (0.11-18.6) | 0.79 |  |  |  |
| Coronary artery disease | 1.67 (0.27-10.3) | 0.58 |  |  |  |
| Pulmonary disease | 0.14 (0.01-1.55) | 0.11 |  |  |  |
| Preoperative eGFR | 1.01 (0.96-1.06) | 0.78 |  |  |  |
| Tumor size (cm) | 0.85 (0.63-1.13) | 0.27 |  |  |  |
| Tumor thrombus level  Level 3  Level 4 | Reference  5.0 (0.72-34.7) | 0.10 |  |  |  |
| Neoadjuvant treatment | 1.67 (0.18-15.1) | 0.65 |  |  |  |
| Tumor necrosis | 4.20 (0.59-30.1) | 0.15 |  |  |  |
| Metastatic disease | 6.00 (0.81-44.4) | 0.08 | 6.51 (0.54-78.4) | 0.14 |  |
